# Supplementary material for: Multivariate Genetic Structure of Externalizing Behavior and Structural Brain Development in a Longitudinal Adolescent Twin Sample
Source: Int J Mol Sci. 2022 Mar 15;23(6):3176. doi: 10.3390/ijms23063176 (PMC8949114; doi:10.3390/ijms23063176)
Supplement: Supplementary file 1 [file ijms-23-03176-s001.zip › ijms-1599281-supplementary.pdf]

Supplementary Materials

S.1. Externalizing behavior on the Child Behavior Checklist and Youth Self Report

S.1.1. Items available on the Child Behavior Checklist and Youth Self Report

Externalizing behavior of the children was assessed by the parent-reported Child Behavior Checklist (CBCL) and Youth Self Report (YSR) [2]. Both questionnaires have similar items for the 'Externalizing' scale (**Supplementary Table S1**). Items 73 (*SexProbs*), 88 (*Sulks*), and 106 (*Vandalism*) on the CBCL were not included on the YSR. The sum scores of the CBCL and YSR can therefore not be directly compared. In addition, the items 2 (*Alcohol*), 28 (*BreaksRules*), and 99 (*Smoking*) on the rule-breaking subscale were not on the 1999 version of CBCL administered to the siblings at baseline assessment. These three items were therefore excluded in the analysis of the CBCL scores at all three assessments, but were not excluded in the analysis of the YSR because no direct comparison is made between the CBCL and the YSR.

Supplementary Table S1. Items on the Child Behavior Checklist and the Youth Self Report.

	**Aggressive behavior scale**		**Rule-breaking behavior scale**
3	Argues a lot	2 [1]	Drinks alcohol without parents' approval
16	Cruelty, bullying, or meanness to others	26	Doesn't seem to feel guilty after misbehaving
19	Demands a lot of attention	28 [1]	Breaks rules at home, school, or elsewhere
20	Destroys his/her own things	39	Hangs around with other who get in trouble
21	Destroys things belonging to his/her family or others	43	Lying or cheating
22	Disobedient at home	63	Prefers being with older kids
23	Disobedient at school	67	Runs away from home
37	Gets in many fights	72	Sets fires
57	Physically attacks people	73 [1]	Sexual problems
68	Screams a lot	81	Steals at home
86	Stubborn, sullen, or irritable	82	Steals outside the home
87	Sudden changes in mood or feelings	90	Swearing or obscene language
88 [1]	Sulks a lot	96	Thinks about sex too much
89	Suspicious	99 [1]	Smokes, chews, sniffs tobacco
94	Teases a lot	101	Truancy, skips school
95	Temper tantrums or hot temper	105	Uses drugs for nonmedical purposes
97	Threatens people	106 [1]	Vandalism
104	Unusually loud		

[1] *item not available on YSR. b item not available on the 1999 version of CBCL administered to the siblings at baseline assessment #1 (age 9 years). Abbreviations (in alphabetical order): CBCL = Child Behavior Check List; YSR = Youth Self Report.*

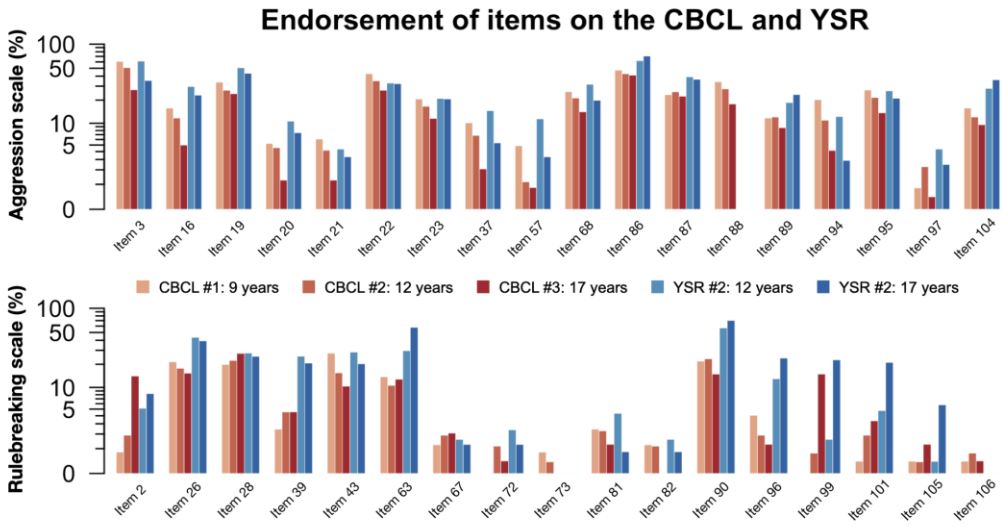

Supplementary Figure S1. Endorsement of individual items on the parent-reported Child Behavior Checklist and the self-report Youth Self Report. A response of '1: occasionally' or '2: often' was considered an endorsement. See **Supplementary Table S1** for description of items. Percentage of endorsement is plotted on a logarithmic scale for visualization purposes only. Abbreviation (in alphabetical order): CBCL = Child Behavior Check List; YSR = Youth Self Report.

S.1.2. Distribution of the summary scores of externalizing behavior on the Child Behavior Checklist and Youth Self Report

The distributions of the sum scores on the CBCL and YSR scales are positively skewed and have inflated zero responses (mean skewness: +1.48; range skewness: +0.96 to +2.20; zero responses: 3% to 36%; **Supplementary Figure S2**). A log-normal distribution described the data better than a normal distribution for most sum scores; therefore, the scores were log_{10}-transformed prior to statistical analysis (**Supplementary Figure S2**).

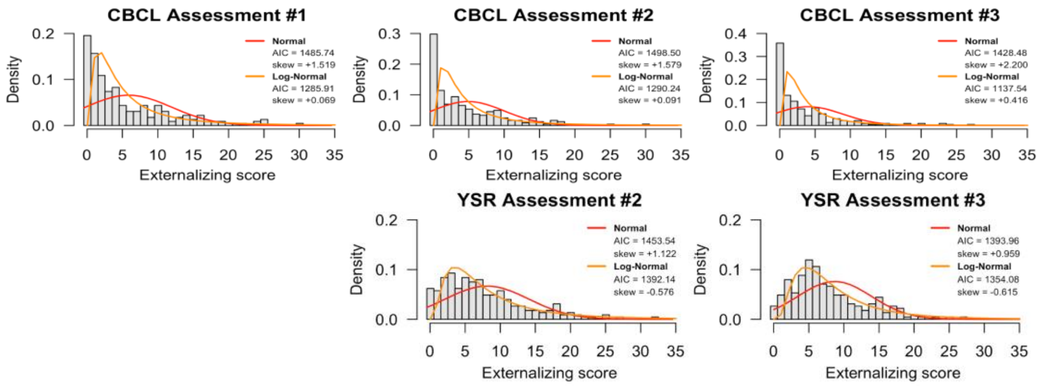

Supplementary Figure S2. Distribution of the scores on the externalizing scale of the CBCL and YSR. A log-normal distribution describes the dispersion of the scores better than a normal distribution and reduces the skewness for all scores. No YSR was available at baseline assessment. Abbreviations (in alphabetical order): CBCL = Child Behavior Check List; YSR = Youth Self Report.

S.2. Genetic modeling of twin and sibling data

S.2.1. Correlations of externalizing behavior between pairs of monozygotic and dizygotic twins and their sibling

The correlation of externalizing behavior between monozygotic twin pairs is on average 1.8x larger than the correlation between dizygotic twin pairs (**Supplementary Table S2**). This supports the high heritability estimates computed by the genetic models (**Table 2**; **Supplementary Table S3**).

Supplementary Table S2. Correlations between members of monozygotic and dizygotic twin pairs for externalizing behavior scales on the Child Behavior Checklist and Youth Self Report.

Trait (Untransformed scores)	MZ twins	DZ twins	Twin-Sibling
CBCL Ext #1	+0.817	+0.441	+0.536
CBCL Ext #2	+0.896	+0.777	+0.459
CBCL Ext #3	+0.718	+0.329	+0.296
YSR Ext #2	+0.768	+0.336	+0.166
YSR Ext #3	+0.176	+0.336	+0.008

Trait (log_{10}-transformed scores)	MZ twins	DZ twins	Twin-Sibling
CBCL Ext #1	+0.910	+0.576	+0.453
CBCL Ext #2	+0.954	+0.676	+0.482
CBCL Ext #3	+0.888	+0.463	+0.365
YSR Ext #2	+0.758	+0.371	+0.163
YSR Ext #3	+0.329	+0.171	+0.099

Assessment #1: age 10 years; #2: age 13 years; #3: age 18 years. Abbreviations (in alphabetical order): CBCL = Child Behavior Check List; DZ = dizygotic; MZ = monozygotic; YSR = Youth Self Report.

S.2.2. Heritability of the untransformed scores of externalizing behavior on the Child Behavior Checklist and Youth Self Report

Heritability analysis of the externalizing scale on the CBCL and YSR were repeated using untransformed scores to address the potential bias introduced by a log_{10}-transformation of the scores because of its multiplicative property [121]. A similar pattern of genetic and environmental influences that exists for the log_{10}-transformed scores (**Table 2** in main text) is also present for the untransformed scores (**Supplementary Table S3**). However, heritability estimates are slightly lower than estimates for the log_{10}-transformed scores (average delta = –18%; range delta = [–37%; –10%]), but remain significant across the board. Estimates for common environment are slightly increased compared to estimates for the log_{10}-transformed scores (average delta = +5%; range delta = [–1%; +18%]), and mostly affects the externalizing scores on the parent-reported CBCL at ages 9 and 12 years that barely reached significance using the untransformed scores (c^2 = 27% and 15% respectively; p~0.031).

Supplementary Table S3. Heritability of externalizing behavior on the Child Behavior Checklist and Youth Self Report from analyzing untransformed scores.

Instrument	Age 10 years	Age 13 years	Age 18 years
CBCL Externalizing	h^2 = **43% [11%; 71%]**	h^2 = **75% [57%; 89%]**	h^2 = **62% [39%; 75%]**
	c^2 = **27% [3%; 50%]**	c^2 = **15% [1%; 32%]**	c^2 = 4% [0%;22%]
	e^2 = 30% [19%; 47%]	e^2 = 11% [7%; 18%]	e^2 = 34% [23%; 49%]
YSR Externalizing		h^2 = **66% [45% ;79%]**	h^2 = **30% [8%; 53%]**
	N/A	c^2 = 0% [0%; 14%]	c^2 = 0% [0%; 17%]
		e^2 = 34% [21%; 55%]	e^2 = 70% [47%; 92%]

*Heritability (h^2), common environment (c^2), unique environment (e^2) and their 95% confidence interval is reported for the longitudinal genetic model on the untransformed scores of the externalizing scale of the CBCL and YSR. Results for the log_{10}-transformed scores are reported in **Table 2** of the main text. Values printed in **boldface** are significant (p < 0.05); exact p-values are reported in **Supplementary Data File F1**. Abbreviations (in alphabetical order): CBCL = Child Behavior Check List; YSR = Youth Self Report.*

S.2.3. Phenotypic and genetic correlations between longitudinal assessments of externalizing behavior on the Child Behavior Checklist and Youth Self Report

A pairwise bivariate genetic model was used to estimate the phenotypic (r_{ph}), genetic (r_a), common environmental (r_c), and unique environmental (r_e) associations between the

externalizing scales on the CBCL and YSR, and between longitudinal assessments of the same instrument at different ages (**Supplementary Table S4**). Summary scores of the questionnaires showed moderate to good phenotypic associations between assessments (range r_{ph} between +0.46 to +0.64; **Supplementary Table S4**), The same genetic factor was in part responsible for the behavior assessed by each scale at different ages during adolescent development (range r_a between +0.44 to +0.98; **Supplementary Table S4**). Judging by the confidence intervals, the genetic factor for the YSR questionnaire between mean ages 13 and 18 years was completely overlapping. In contrast, the genetic factors for the CBCL questionnaire are only partially overlapping at the different ages, suggesting the presence of unique genetic factors specific for each age group.

Supplementary Table S4. Phenotypic, genetic and environmental correlations between longitudinal assessments of externalizing behavior on the Child Behavior Checklist and Youth Self Report.

Instrument	Age 10 -> 13 years	Age 13 -> 18 years	Age 10 -> 18 years
CBCL Externalizing	r_{ph} = **+0.63 [+0.53; +0.72]**	r_{ph} = **+0.58 [+0.47; +0.68]**	r_{ph} = **+0.51 [+0.38; +0.63]**
	r_a = **+0.63 [+0.62; +0.76]**	r_a = **+0.59 [+0.50; +0.71]**	r_a = **+0.53 [+0.45; +0.71]**
	r_c = +1.00 [–1.00; +1.00]	r_c = +1.00 [–1.00 ;+1.00]	r_c = +1.00 [–1.00; +1.00]
	r_e = +0.43 [–0.03; +0.71]	r_e = +0.31 [–0.03; +0.58]	r_e = +0.16 [–0.26; +0.52]
YSR Externalizing	N/A	r_{ph} = **+0.46 [+0.32; +0.57]**	N/A
		r_a = **+0.86 [+0.57; +1.00]**	
		r_c = +0.58 [–1.00; +1.00]	
		r_e = –0.08 [–0.40; +0.26]	

*Phenotypic correction (r_{ph}), genetic correlation (r_a), common environmental correlation (r_c) and unique environmental correlation (r_e) between assessments and their 95% confidence interval is reported for the longitudinal genetic model on the log_{10}-transformed scores of the externalizing scale of the CBCL and YSR. Values printed in **boldface** are significant (p < 0.05); exact p-values are reported in **Supplementary Data File F1**. Abbreviations (in alphabetical order): CBCL = Child Behavior Check List; YSR = Youth Self Report.*

S.3. Magnetic resonance Imaging measures of the brain

S.3.1. Cortical and subcortical regions previously implicated in externalizing behavior

Gray matter volumes of cortical and subcortical brain structures were obtained from Freesurfer Desikan-Killiany atlas and Aseg atlas [112]. We limit ourselves to subcortical structures and cortical regions on the frontal, temporal, and insular cortex based on previous research (**Supplementary Figure S3**; **Supplementary Table S5**) [55–56,60,103,114].

Supplementary Table S5. List of cortical and subcortical gray matter volumes from FreeSurfer atlas.

ID	Abbreviation	Region
1/2	SFG [L/R]	Superior frontal gyrus
3/4	cMFG [L/R]	Caudal middle frontal gyrus
5/6	rMFG [L/R]	Rostral middle frontal gyrus
7/8	FP [L/R]	Frontal pole
9/10	mOFG [L/R]	Medial orbitofrontal gyrus
11/12	lOFG [L/R]	Lateral orbitofrontal gyrus
13/14	Opr [L/R]	Pars opercularis
15/16	Orb [L/R]	Pars orbitalis
17/18	Tri [L/R]	Pars triangularis
19/20	TTG [L/R]	Transverse temporal gyrus
21/22	STG [L/R]	Superior temporal gyrus
23/24	Bank [L/R]	Banks of superior temporal gyrus
25/26	MTG [L/R]	Middle temporal gyrus
27/28	TP [L/R]	Temporal pole

29/30	ITG [L/R]	Inferior temporal gyrus
31/32	Ent [L/R]	Entorhinal gyrus
33/34	FG [L/R]	Fusiform gyrus
35/36	Par [L/R]	Parahippocampal gyrus
37/38	ACc [L/R]	Caudal anterior cingulate gyrus
39/40	ACr [L/R	Rostral anterior cingulate gyrus
41/42	Ins [L/R]	Insula
43/44	AMY [L/R]	Amygdala
45/46	HIP [L/R]	Hippocampus
47/48	PUT [L/R]	Putamen

Abbreviations (in alphabetical order): Abbr = abbreviation; L = left; R = right.

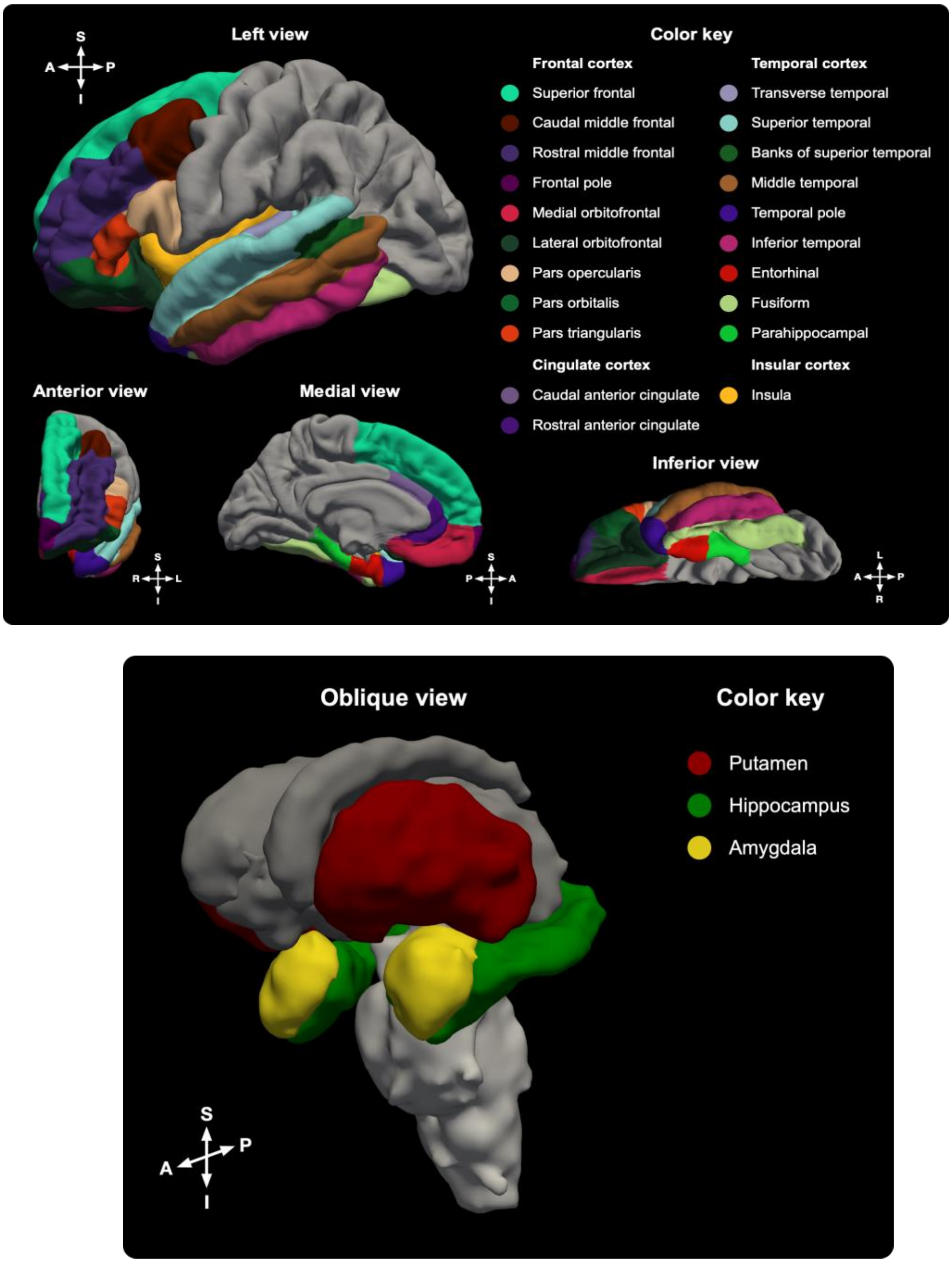

Supplementary Figure S3. A 3D representation of the cortical regions on the frontal, temporal and insular cortex and subcortical structures of the brain used in the analyses. Abbreviation (in alphabetical order): A = anterior; I = inferior; L = left; P = posterior; S = superior.

S.3.2. Major white matter tracts of the human brain in the John Hopkins University atlas

The John Hopkins University (JHU) white matter atlas provides manual annotation for 20 major white matter tracts (**Supplementary Table S6**; **Supplementary Figure S4**). The atlas consists of 9 bilateral tracts and 2 commissural tracts of the brain. A global brain measure is defined by the region of all white matter tracts combined.

Supplementary Table S6. List of the major white matter tracts in the John Hopkins University atlas.

ID	Abbreviation	Full name	Volume [mm³]
1/2	ATR [L/R]	Anterior thalamic radiation	8706 / 8014
3/4	CST [L/R]	Corticospinal tract	5727 / 5012
5/6	CGC [L/R]	Cingulum (cingulate gyrus)	1903 / 974
7/8	CGH [L/R]	Cingulum (hippocampus)	557 / 798
9/10	IFOF [L/R]	Inferior fronto-occipital fasciculus	5794 / 6957
11/12	ILF [L/R]	Inferior longitudinal fasciculus	5826 / 3725
13/14	SLF [L/R]	Superior longitudinal fasciculus	9870 / 7787
15/16	SLFtemp [L/R]	Superior longitudinal fasciculus (temporal part)	76 / 298
17/18	UNC [L/R]	Uncinate fasciculus	1201 / 784
19	FMajor	Forceps major	6458
20	FMinor	Forceps minor	19407
–	Global	All major white matter tracts combined	99874

Abbreviations (in alphabetical order): Abbr = abbreviation; L = left; R = right.

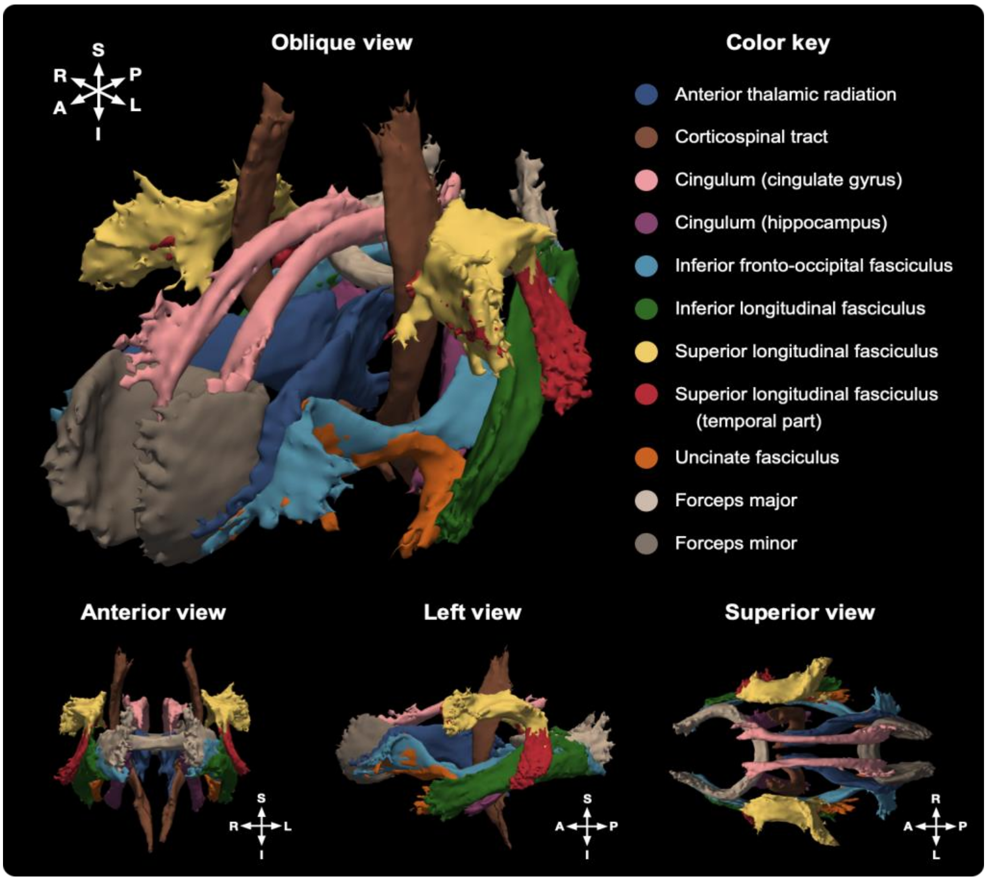

Supplementary Figure S4. A 3D representation of the major white matter tracts in the John Hopkins University atlas.

S.3.3. *Heritability of global brain development during adolescence*

Heritability for the six global brain measures is generally high (h^2 range: 34% to 93%; **Supplementary Table S7**), and influences from common environment shared between family members only significant for global mean diffusivity (c^2 range: 10% to 28%; **Supplementary Table S7**). Longitudinal change rates in total brain volume between all ages, and global mean diffusivity between ages 10 to 13 years are also heritable (h^2_Δ range: 9% to 64%; **Supplementary Table S8**). In addition, longitudinal change rates in global mean diffusivity between all ages is influenced by the common environment (c^2_Δ range: 35% to 55%; **Supplementary Table S8**).

Supplementary Table S7. Heritability estimates of global brain measures throughout adolescence.

Brain structure	Age 10 years	Age 13 years	Age 18 years
Total brain volume	h^2 = **89% [75%; 94%]**	h^2 = **92% [79%; 95%]**	h^2 = **93% [77%; 97%]**
	c^2 = 2% [0%; 16%]	c^2 = 1% [0%; 14%]	c^2 = 3% [0%; 20%]
	e^2 = 8% [6%; 13%]	e^2 = 8% [5%; 12%]	e^2 = 4% [2%; 6%]
Total cortical gray matter volume [1]	h^2 = **67% [49%; 78%]**	h^2 = **49% [26%; 77%]**	h^2 = **67% [44%; 78%]**
	c^2 = 0% [0%; 14%]	c^2 = 20% [0%; 42%]	c^2 = 3% [0%; 25%]
	e^2 = 32% [21%; 45%]	e^2 = 31% [21%; 43%]	e^2 = 30% [21%; 41%]
Total subcortical gray matter volume [1]	h^2 = **61% [38%; 74%]**	h^2 = **59% [32%; 80%]**	h^2 = **63% [37%; 83%]**
	c^2 = 5% [0%; 22%]	c^2 = 14% [0%; 36%]	c^2 = 14% [0%; 35%]
	e^2 = 34% [24%; 47%]	e^2 = 27% [18%; 40%]	e^2 = 23% [16%; 35%]
Total cerebral white matter volume [1]	h^2 = **68% [52%; 78%]**	h^2 = **56% [36%; 79%]**	h^2 = **72% [46%; 87%]**
	c^2 = 1% [0%; 12%]	c^2 = 19% [0%; 38%]	c^2 = 9% [0%; 32%]
	e^2 = 31% [21%; 45%]	e^2 = 26% [18%; 36%]	e^2 = 19% [12%; 28%]
Global mean fractional anisotropy	h^2 = **64% [43%; 76%]**	h^2 = **73% [44%; 81%]**	h^2 = **51% [21%; 78%]**
	c^2 = 2% [0%; 16%]	c^2 = 0% [0%; 26%]	c^2 = 18% [0%; 41%]
	e^2 = 34% [23%; 49%]	e^2 = 27% [19%; 39%]	e^2 = 31% [20%; 46%]
Global mean diffusivity	h^2 = **73% [53%; 85%]**	h^2 = **69% [44%; 84%]**	h^2 = **34% [13%; 64%]**
	c^2 = **10% [1%; 29%]**	c^2 = **10% [0%; 31%]**	c^2 = **28% [6%; 46%]**
	e^2 = 16% [11%; 24%]	e^2 = 21% [14%; 32%]	e^2 = 38% [25%; 52%]

[1] Following regression of total brain volume.

Heritability or common environmental estimates printed in ***boldface*** *are significant ($p < 0.05$); exact p-values are reported in* ***Supplementary Data File F2****. Abbreviations (in alphabetical order): h^2 = heritability, or proportion of phenotypic variation attributed to additive genetic influences; c^2 = proportion of phenotypic variation attributed to common environmental influences; e^2 = proportion of phenotypic variation attributed to unique environmental influences.*

Supplementary Table S8. Heritability estimates of longitudinal changes in global brain measures throughout adolescence.

Brain structure	Age 10 -> 13 years	Age 13 -> 18 years	Age 10 -> 18 years
Total brain volume	h^2_Δ = **33% [7%; 59%]**	h^2_Δ = **35% [4%; 65%]**	h^2_Δ = **64% [40%; 77%]**
	c^2_Δ = 3% [0%; 22%]	c^2_Δ = 9% [0%; 34%]	c^2_Δ = 1% [0%; 15%]
	e^2_Δ = **64% [41%; 86%]**	e^2_Δ = **55% [35%; 78%]**	e^2_Δ = **35% [23%; 52%]**
Total cortical gray matter volume [1]	h^2_Δ = 3% [0%; 38%]	h^2_Δ = 3% [0%; 37%]	h^2_Δ = 0% [0%; 21%]
	c^2_Δ = 15% [0%; 28%]	c^2_Δ = 13% [0%; 30%]	c^2_Δ = 1% [0%; 9%]
	e^2_Δ = **82% [58%; 96%]**	e^2_Δ = **84% [59%; 97%]**	e^2_Δ = **99% [79%; 100%]**
Total subcortical gray matter volume [1]	h^2_Δ = 4% [0%; 32%]	h^2_Δ = 33% [0%; 56%]	h^2_Δ = 10% [0%; 34%]
	c^2_Δ = 7% [0%; 24%]	c^2_Δ = 0% [0%; 34%]	c^2_Δ = 6% [0%; 21%]
	e^2_Δ = **89% [66%; 100%]**	e^2_Δ = **67% [43%; 94%]**	e^2_Δ = **84% [64%; 98%]**
Total cerebral white matter volume [1]	h^2_Δ = 2% [0%; 32%]	h^2_Δ = 27% [0%; 50%]	h^2_Δ = 19% [0%; 45%]
	c^2_Δ = 12% [0%; 26%]	c^2_Δ = 1% [0%; 26%]	c^2_Δ = 5% [0%; 24%]
	e^2_Δ = **86% [64%; 99%]**	e^2_Δ = **72% [50%; 94%]**	e^2_Δ = **76% [53%; 95%]**

Global mean fractional anisotropy	h^2_{Δ} = 0% [0%; 13%]<br>c^2_{Δ} = 1% [0%; 10%]<br>**e^2_{Δ} = 99% [86%; 100%]**	h^2_{Δ} = 13% [0%; 53%]<br>c^2_{Δ} = 23% [0%; 46%]<br>**e^2_{Δ} = 65% [44%; 85%]**	h^2_{Δ} = 8% [0%; 38%]<br>c^2_{Δ} = 13% [0%; 29%]<br>**e^2_{Δ} = 79% [58%; 93%]**
Global mean diffusivity	**h^2_{Δ} = 9% [1%; 34%]**<br>**c^2_{Δ} = 55% [33%; 68%]**<br>**e^2_{Δ} = 35% [25%; 47%]**	h^2_{Δ} = 2% [0%; 28%]<br>**c^2_{Δ} = 47% [24%; 60%]**<br>**e^2_{Δ} = 52% [37%; 66%]**	h^2_{Δ} = 13% [0%; 53%]<br>**c^2_{Δ} = 35% [6%; 53%]**<br>**e^2_{Δ} = 52% [34%; 67%]**

[1] Following regression of total brain volume.

*Heritability or common or unique environmental estimates of the longitudinal change scores printed in **boldface** are significant ($p < 0.05$); exact p-values are reported in **Supplementary Data File F2**. Abbreviations (in alphabetical order): h^2_{Δ} = heritable of longitudinal change rates; c^2_{Δ} = proportion of common environmental influences on longitudinal change rates; e^2_{Δ} = proportion of unique environmental influences on longitudinal change rate.*

S.3.4. Longitudinal development of brain measures during adolesence

Longitudinal development of gray matter volume of regional (sub)cortical brain structures and mean fractional anisotropy (FA) and mean diffusivity (MD) of the major white matter tracts (**Supplementary Figure S5**; **Supplementary Figure S6**). Exact values are reported in **Supplementary Data File F2**.

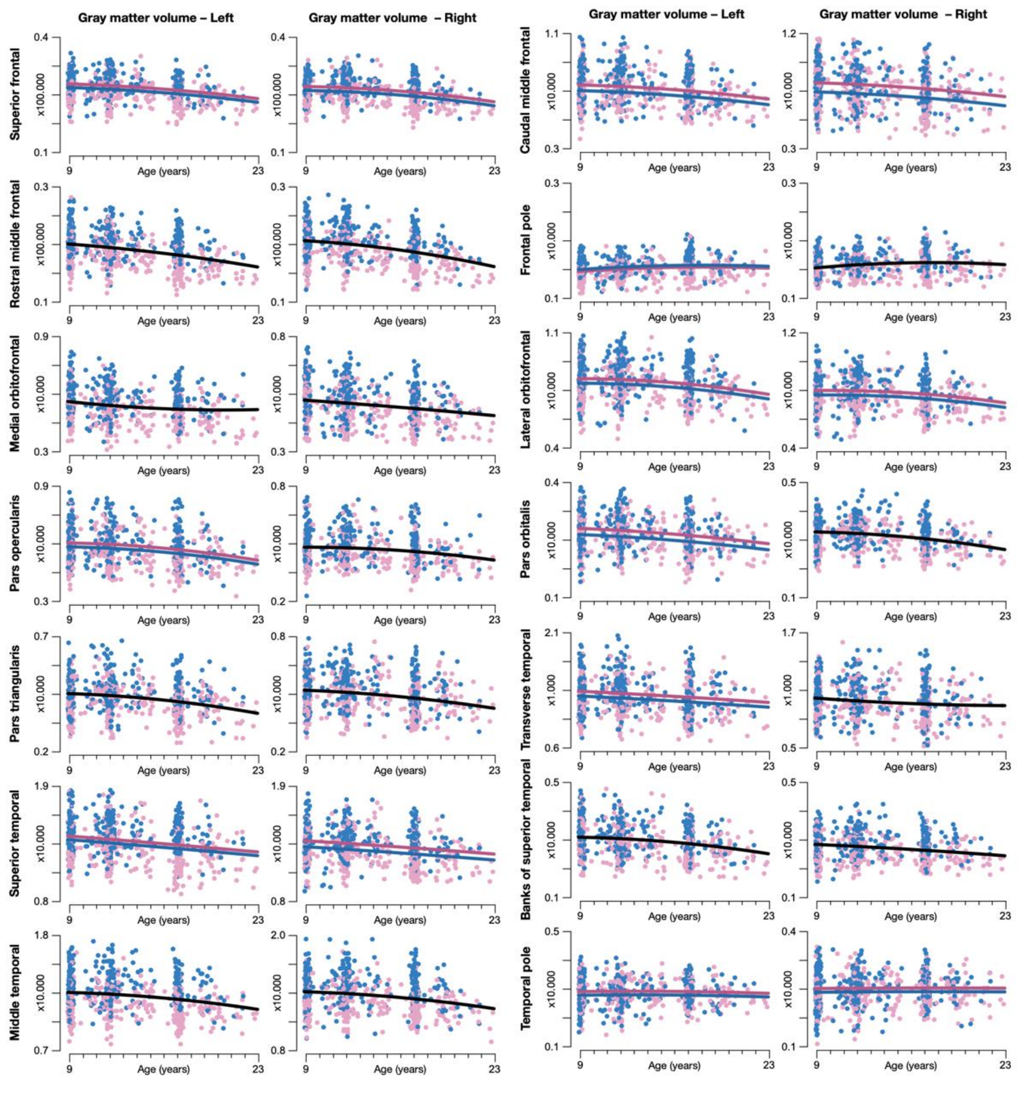

Supplementary Figure S5 (continued on next page). Development of cortical and subcortical gray matter volumes. Abbreviation (in alphabetical order): CI = confidence interval; n.s. = not significant.

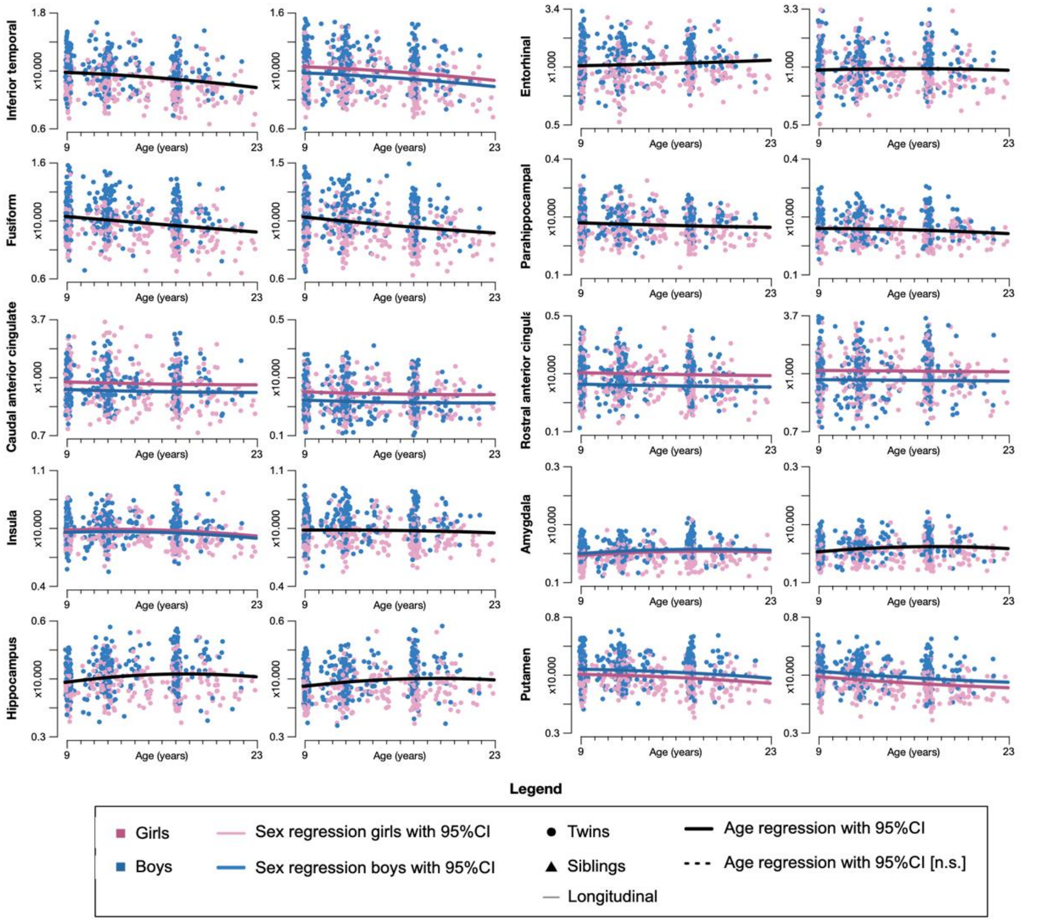

Supplementary Figure S5 (continued from previous page). Development of cortical and subcortical gray matter volumes. Abbreviation (in alphabetical order): CI = confidence interval; n.s. = not significant.

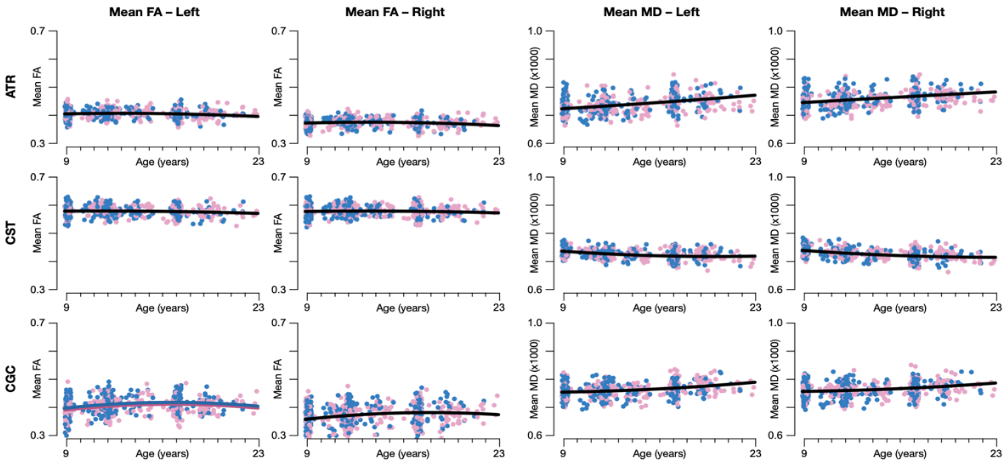

Supplementary Figure S6 (continued on next page). Development of mean fractional anisotropy and mean diffusivity of the major white matter tracts. Abbreviations (in alphabetical order): ATR = anterior thalamic radiation; CGC = cingulum (cingulate gyrus); CGH = cingulum (hippocampal); CI = confidence interval; CST = corticospinal tract; FA = fractional anisotropy; Fmajor = forceps major; Fminor = forceps minor; ILF = inferior longitudinal fasciculus; IFOF = inferior fronto-occipital fasciculus; MD = mean diffusivity; SLF = superior longitudinal fasciculus; SLFtemp = superior longitudinal fasciculus (temporal part); UNC = uncinate fasciculus.

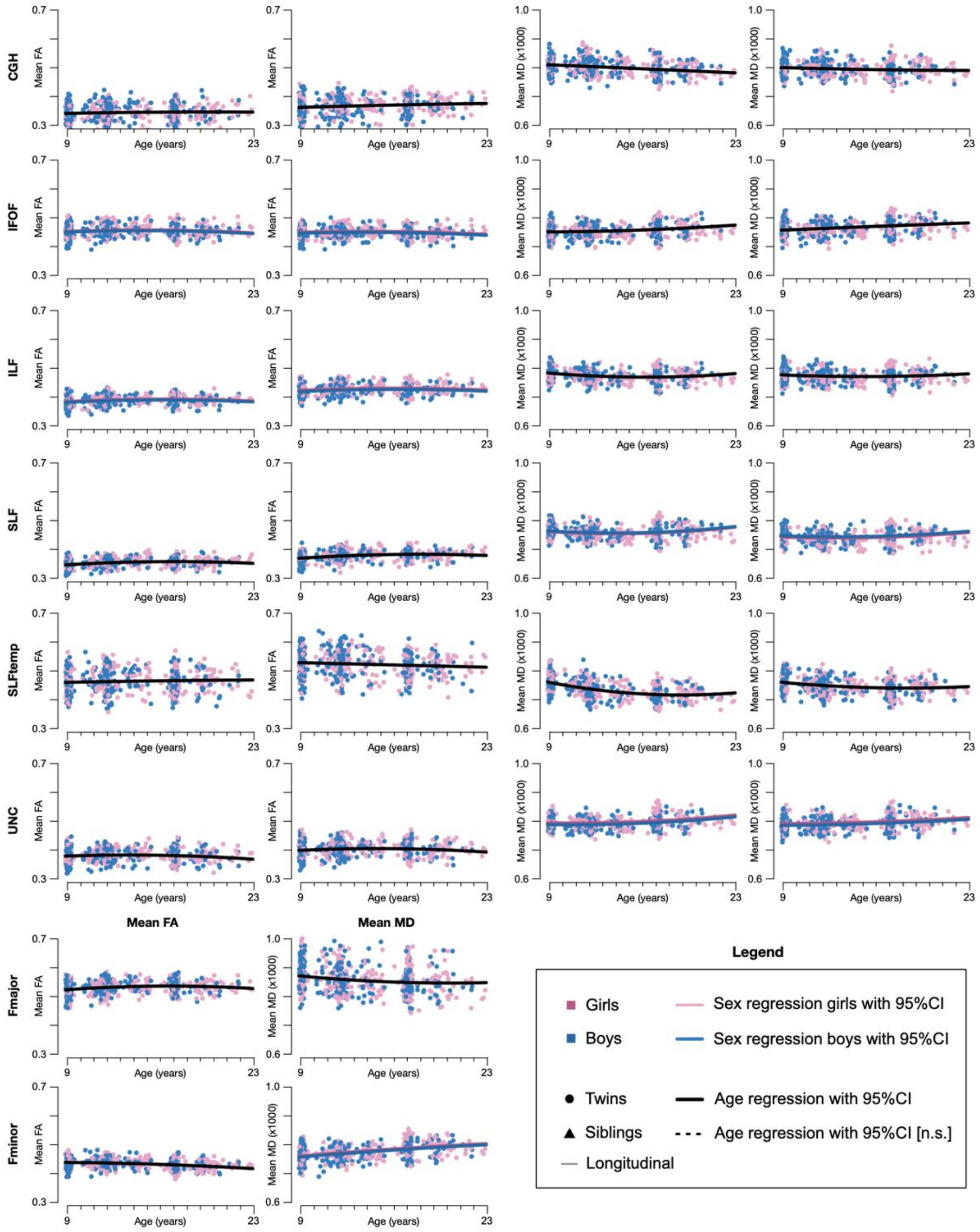

Supplementary Figure S6 (continued from previous page). Development of mean fractional anisotropy and mean diffusivity of the major white matter tracts. Abbreviations (in alphabetical order): ATR = anterior thalamic radiation; CGC = cingulum (cingulate gyrus); CGH = cingulum (hippocampal); CI = confidence interval; CST = corticospinal tract; FA = fractional anisotropy; Fmajor = forceps major; Fminor = forceps minor; ILF = inferior longitudinal fasciculus; IFOF = inferior fronto-occipital fasciculus; MD = mean diffusivity; SLF = superior longitudinal fasciculus; SLFtemp = superior longitudinal fasciculus (temporal part); UNC = uncinate fasciculus.

S.4. Associations between externalizing behavior and global brain structures

The associations between brain structures and externalizing behavior were sparse and occurred mainly during mid-adolescence, around age 13 years, although the direction and magnitude of the associations were overall consistent throughout adolescence (**Supplementary Figure S7**; **Supplementary Table S9**; **Supplementary Data File F3**)

Supplementary Table S9. Phenotypic, genetic, and environmental associations between global brain measures and externalizing behavior.

Brain structure vs. CBCL Externalizing		Age 10 years	Age 13 years	Age 18 years
Total brain volume	r_{ph} =	−0.06 [−0.21; +0.09]	−0.11 [−0.25; +0.04]	−0.06 [−0.20; +0.09]
	r_a =	−0.11 [−0.30; +0.04]	−0.12 [−0.28; +0.05]	−0.07 [−0.24; +0.13]
	r_c =	+0.74 [−1.00; +1.00]	+0.56 [−1.00; +1.00]	+0.36 [−0.93; +1.00]
	r_e =	−0.14 [−0.50; +0.25]	−0.22 [−0.53; +0.15]	−0.17 [−0.48; +0.18]
Total cortical gray matter volume [1]	r_{ph} =	−0.02 [−0.17; +0.12]	**−0.20 [−0.35; −0.04]**	+0.07 [−0.08; +0.21]
	r_a =	+0.08 [−0.18; +0.39]	−0.01 [−0.04; +0.02]	+0.18 [−0.05; +0.42]
	r_c =	−0.91 [−1.00; +1.00]	**−0.97 [−1.00; −0.27]**	−0.94 [−1.00; +1.00]
	r_e =	+0.06 [−0.34; +0.44]	−0.12 [−0.41; +0.18]	+0.14 [−0.20; +0.42]
Total subcortical gray matter volume [1]	r_{ph} =	−0.05 [−0.20; +0.10]	−0.05 [−0.20; +0.10]	−0.05 [−0.20; +0.10]
	r_a =	−0.11 [−0.42; +0.17]	−0.08 [−0.35; +0.07]	−0.07 [−0.35; +0.19]
	r_c =	+0.01 [−1.00; +1.00]	+0.12 [−1.00; +1.00]	−0.33 [−1.00; +1.00]
	r_e =	+0.12 [−0.23; +0.42]	−0.03 [−0.33; +0.27]	+0.17 [−0.16; +0.45]
Total cerebral white matter volume [1]	r_{ph} =	+0.03 [−0.11; +0.18]	+0.15 [−0.00; +0.31]	−0.09 [−0.24; +0.06]
	r_a =	−0.05 [−0.40; +0.21]	+0.01 [−0.24; +0.26]	−0.19 [−0.41; +0.07]
	r_c =	+0.98 [−1.00; +1.00]	+0.93 [−1.00; +1.00]	+0.68 [−1.00; +1.00]
	r_e =	−0.06 [−0.46; +0.35]	+0.08 [−0.21; +0.38]	−0.08 [−0.41; +0.25]
Global mean fractional anisotropy	r_{ph} =	**+0.18 [+0.02; +0.32]**	**+0.20 [+0.04; +0.35]**	−0.01 [−0.16; +0.15]
	r_a =	+0.09 [−0.25; +0.37]	+0.10 [−0.16; +0.34]	+0.01 [−0.33; +0.30]
	r_c =	+0.77 [−1.00; +1.00]	+0.98 [−1.00; +1.00]	−0.26 [−1.00; +1.00]
	r_e =	+0.23 [−0.18; +0.57]	+0.06 [−0.25; +0.35]	+0.13 [−0.20; +0.43]
Global mean diffusivity	r_{ph} =	−0.13 [−0.28; +0.03]	**−0.20 [−0.35; −0.04]**	+0.04 [−0.11; +0.20]
	r_a =	−0.23 [−0.50; +0.04]	**−0.27 [−0.48; −0.17]**	−0.12 [−0.51; +0.23]
	r_c =	+0.32 [−1.00; +1.00]	+0.29 [−1.00; +1.00]	+0.87 [−1.00; +1.00]
	r_e =	−0.03 [−0.35; +0.30]	−0.19 [−0.48; +0.13]	−0.12 [−0.41; +0.20]

Brain structure vs. YSR Externalizing		Age 10 years	Age 13 years	Age 18 years
Total brain volume	r_{ph} =	N/A	−0.13 [−0.27; +0.02]	−0.02 [−0.16; +0.12]
	r_a =		−0.14 [−0.33; +0.08]	−0.07 [−0.27; +0.25]
	r_c =		+1.00 [−1.00; +1.00]	+1.00 [−1.00; +1.00]
	r_e =		−0.22 [−0.55; +0.18]	−0.07 [−0.38; +0.26]
Total cortical gray matter volume [1]	r_{ph} =	N/A	**−0.17 [−0.32; −0.01]**	+0.05 [−0.10; +0.19]
	r_a =		−0.20 [−0.59; +0.13]	+0.08 [−0.61; +0.58]
	r_c =		+0.98 [−1.00; +1.00]	−1.00 [−1.00; +1.00]
	r_e =		−0.12 [−0.46; +0.24]	+0.04 [−0.25; +0.33]
Total subcortical gray matter volume [1]	r_{ph} =	N/A	−0.07 [−0.22; +0.08]	−0.13 [−0.27; +0.02]
	r_a =		−0.14 [−0.60; −0.14]	−0.08 [−0.73; +0.59]
	r_c =		−0.73 [−1.00; +1.00]	−1.00 [−1.00; +1.00]
	r_e =		+0.19 [−0.14; +0.50]	+0.02 [−0.28; +0.31]
Total cerebral white matter volume [1]	r_{ph} =	N/A	**+0.20 [+0.04; +0.35]**	−0.00 [−0.14; +0.14]
	r_a =		+0.22 [−0.16; +0.67]	+0.02 [−0.47; +0.65]
	r_c =		+0.81 [−1.00; +1.00]	−0.66 [−1.00; +1.00]
	r_e =		+0.16 [−0.22; +0.50]	+0.02 [−0.28; +0.31]
Global mean fractional anisotropy	r_{ph} =	N/A	**+0.18 [+0.02; +0.33]**	+0.13 [−0.02; +0.27]
	r_a =		**+0.42 [+0.42; +0.99]**	+0.32 [−0.43; +1.00]
	r_c =		+0.74 [−1.00; +1.00]	−0.19 [−1.00; +1.00]
	r_e =		−0.36 [−0.62; +0.00]	−0.01 [−0.32; +0.31]

Global mean diffusivity	r_{ph} =	N/A	**–0.20 [–0.34; –0.04]**	**–0.20 [–0.34; –0.05]**
	r_a =		**–0.33 [–0.77; –0.28]**	–0.56 [–0.99; +0.00]
	r_c =		–0.29 [–1.00; +1.00]	+0.76 [–1.00; +1.00]
	r_e =		+0.22 [–0.16; +0.54]	+0.04 [–0.23; +0.30]

[1] After regression of total brain volume.

Values printed in ***boldface*** *were significant (p<0.05); exact p-values are reported in* ***Supplementary Data File F3****. Abbreviations (in alphabetical order): CBCL = Child Behavior Check List; r_{ph} = phenotypic association; r_a = genetic association; r_c = common environment association; r_e = unique environment association; YSR = Youth Self Report.*

S.4.1. Assocations between externalizing behavior and regional brain measures

S.4.1.1. Phenotypic associations

Most uncorrected significant associations were seen during mid-adolescence for both CBCL (at age 10: 9/88; at age 13: 26/88; at age 18: 2/88) and for YSR (at age 13: 23/88; at age 18: 13/88), generally featuring the same direction of association as the corresponding global brain structure, with a majority of the associations related to white matter integrity (variance explained R^2 up to 9%; **Supplementary Figure S7**; **Supplementary Data File F3**). In addition, twelve phenotypic associations between the longitudinal change rates of externalizing behavior and change rate in brain measures were significant prior to correction for multiple testing (R^2 up to 5%; **Supplementary Data File F3**). However, none of the associations at the regional level survived corrections for multiple comparisons.

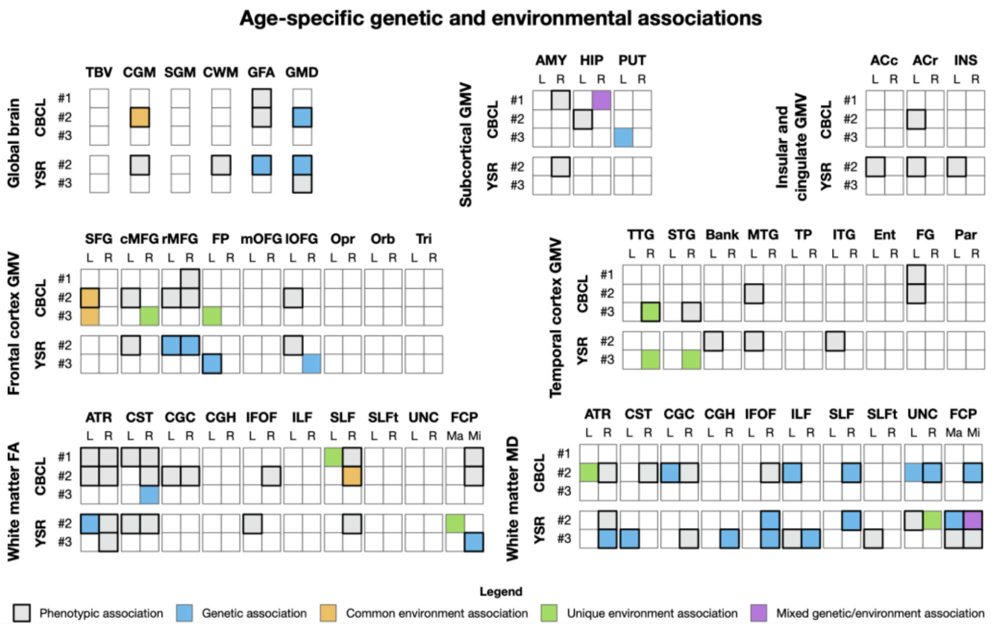

Supplementary Figure S7. Overview of phenotypic, genetic, and environmental associations between brain measures and externalizing behavior. All brain measures except for total brain volume (TBV), global fractional anisotropy (GFA) and global mean diffusivity (GMD) were corrected for the effect of the corresponding global brain measure. Reported associations were significant at uncorrected $p < 0.05$; see **Supplementary Data File F3**. Abbreviations (in alphabetical order): CBCL = Child Behavior Check List; FA = fractional anisotropy; GMV = gray matter volume; L = left; MD = mean diffusivity; R = right; YSR = Youth Self Report; see **Supplementary Table S5** and **Supplementary Table S6** for the abbreviations used for the brain structures.

S.4.1.2. Genetic associations

At a regional level, a shared genetic factor between externalizing behavior and regional brain measures was seen primarily for regional white matter diffusivity (r_a ranging from –0.23 to –0.78; uncorrected $p < 0.05$; **Supplementary Figure S7**; **Supplementary Data File F3**). In addition, of the twelve phenotypic associations between longitudinal change

rates of externalizing behavior and brain measures that were significant prior to correction for multiple testing, only white matter diffusivity of the right superior longitudinal fasciculus had a shared genetic factor with externalizing behavior on the CBCL for their longitudinal change rates between ages 13 and 18 years (r_a = –0.60; uncorrected p = 0.044; **Supplementary Data File F3**), and only gray matter volume of the left pars orbitalis on the frontal cortex had a shared genetic factor with externalizing behavior on the CBCL for their longitudinal change rates between ages 10 and 18 years (r_a = +0.78; uncorrected p = 0.017; **Supplementary Data File F3**).

S.4.1.3. Environmental associations

Several environmental associations between externalizing behavior and regional brain measures were significant prior to correction for multiple testing, primarily at age 13 years (**Supplementary Data File F3**). Influences from common environment shared between the twins and siblings of the same family partially explained the associations of externalizing behavior on the CBCL with gray matter volume of the left superior frontal gyrus (r_c = –0.99; uncorrected p = 0.028) and with white matter fractional anisotropy of the right superior longitudinal fasciculus (r_c = +0.90; uncorrected p = 0.041) at age 13 years. Four phenotypic associations were partially due to influences from the same environmental factors unique to each individual: those between externalizing behavior on the CBCL and right transverse temporal gyrus (r_e = +0.31; uncorrected p = 0.031), between externalizing behavior on the YSR and white matter mean diffusivity of the forceps major tract at age 13 years (r_e = +0.39; uncorrected p = 0.045), between externalizing behavior on the YSR and cortical gray matter volume of the left frontal pole at 18 years (r_e = +0.24; uncorrected p = 0.045), and between longitudinal change rates of externalizing behavior on the CBCL and subcortical gray matter volume of the left putamen between ages 13 and 18 years (r_e = +0.37; uncorrected p = 0.034).

S.5. Supplementary data files

S.5.1. Supplementary Data File F1

The **Supplementary Data File F1** contains four sheets with the phenotypic summary statistics, the heritability estimates, the longitudinal phenotypic, genetic, and environmental associations, and the heritability of the longitudinal change scores of externalizing behavior on the Child Behavior Checklist and the Youth Self Report questionnaire.

S.5.2. Supplementary Data File F2

The **Supplementary Data File F2** contains four sheets with the phenotypic summary statistics, the heritability estimates, the longitudinal phenotypic, genetic, and environmental associations, and the heritability of the longitudinal change scores of the global and regional brain measures.

S.5.3. Supplementary Data File F3

The **Supplementary Data File F3** contains four sheets with the phenotypic, genetic, and environmental associations between externalizing behavior on the Child Behavior Checklist or Youth Self Report with the global or regional brain measures, and phenotypic, genetic, and environmental associations between the longitudinal change scores in externalizing behavior and brain measures.
